# A comparison of methods for interpreting random forest models of genetic association in the presence of non-additive interactions

**DOI:** 10.1186/s13040-021-00243-0

**Published:** 2021-01-29

**Authors:** Alena Orlenko, Jason H. Moore

**Affiliations:** Institute for Biomedical Informatics, University of Pennsylvania, Philadelphia, PA USA

**Keywords:** Machine learning, Feature importances, Random forest, Epistasis, Simulation, Alzheimer’s disease, Glaucoma

## Abstract

**Background:**

Non-additive interactions among genes are frequently associated with a number of phenotypes, including known complex diseases such as Alzheimer’s, diabetes, and cardiovascular disease. Detecting interactions requires careful selection of analytical methods, and some machine learning algorithms are unable or underpowered to detect or model feature interactions that exhibit non-additivity. The Random Forest method is often employed in these efforts due to its ability to detect and model non-additive interactions. In addition, Random Forest has the built-in ability to estimate feature importance scores, a characteristic that allows the model to be interpreted with the order and effect size of the feature association with the outcome. This characteristic is very important for epidemiological and clinical studies where results of predictive modeling could be used to define the future direction of the research efforts. An alternative way to interpret the model is with a permutation feature importance metric which employs a permutation approach to calculate a feature contribution coefficient in units of the decrease in the model’s performance and with the Shapely additive explanations which employ cooperative game theory approach. Currently, it is unclear which Random Forest feature importance metric provides a superior estimation of the true informative contribution of features in genetic association analysis.

**Results:**

To address this issue, and to improve interpretability of Random Forest predictions, we compared different methods for feature importance estimation in real and simulated datasets with non-additive interactions. As a result, we detected a discrepancy between the metrics for the real-world datasets and further established that the permutation feature importance metric provides more precise feature importance rank estimation for the simulated datasets with non-additive interactions.

**Conclusions:**

By analyzing both real and simulated data, we established that the permutation feature importance metric provides more precise feature importance rank estimation in the presence of non-additive interactions.

**Supplementary Information:**

The online version contains supplementary material available at 10.1186/s13040-021-00243-0.

## Background

Machine learning has become a common analytical approach for modeling the relationship between measures of biological systems and clinical outcomes. Models generated from machine learning can be used for prediction, in which case the biological basis for the pattern being modeled may not be of interest. However, biological interpretation of machine learning models can be extremely important if the goal is to generate biological hypotheses that need to be validated clinically or experimentally. As such, methods for model interpretation have become an important component of machine learning research. A group of methods provide a graphical description of the model’s global behavior (i.e. partial dependency plots [1] and decision tree surrogate models). Several methods such as individual conditional expectation (ICE) plots [2], local interpretable model-agnostic explanations or LIME [3] and Shapley additive explanations or SHAP [4] focus on explaining individual model predictions. Another class of methods focus on assigning weights to individual variables or features based on how much information they provide to the predictions being made by the machine learning model. This latter approach generates ‘feature importance scores’ that can be used to create a list of features ranked according to their importance. This allows the modeler to focus on the most important features for biological interpretation. Interpretability (along with performance) is the key quality of the machine learning model, specifically when it is applied to biomedical research goals such as biomarkers discovery and patient diagnostics.

It has been widely discussed that for the complex biomedical phenotypes non-additive epistatic interactions between genes could be present more frequently than previously thought [5–7]. Indeed, gene-gene interactions have been detected in multiple genome-wide association studies of various disease phenotypes, including Alzheimer’s disease [8], cataracts [9], diabetes [10, 11], cardiovascular diseases [12, 13], neurological diseases [14, 15], and various cancer types [16, 17]. Epistasis has been defined in several different ways [18, 19]. Here, we define epistasis as interactions between two or more gene loci such that the phenotype cannot be accurately predicted by simply adding the effects of individual gene loci. This is a statistical definition of epistasis as it measures the deviation from additivity using models that summarize genotypic and phenotypic variability of human population data. In contrast to that, biological epistasis is identified at the cellular level in an individual as a result of a physical interactions among molecules within the biological network. The relationship between two epistatic concepts is complicated such that statistical epistasis does not necessarily translate into biological epistasis [18].

The intrinsic complexity of non-additive interactions creates several analytical and practical challenges for its detection via traditional statistical methods due to its inability to detect non-additive effects in the large volume of data from GWAS studies. Machine learning methods have more flexibility in their power to detect an underlying complexity of genetic architecture and, therefore, has been widely used in epistasis discovery [20]. A large body of research has been accumulated on epistatic interaction detection with neural networks, support vector machines, multifactor dimensionality reduction, and random forest (RF) models [21, 22]. Specifically, RF algorithm is known for its ability to take into account non-additive effects through its hierarchical tree-based structure [20, 23]. A number of studies have been conducted on the integration of RF method into the epistatic interaction discovery. Among them RF with sliding window sequential forward feature selection method [24], mixed RF approach that accounts for both population structure and epistatic interactions [25], relative recurrency variable importance metric (r2VIM) [26] for RF that generates variable set with main and interaction effect, and the permuted RF method that identifies interacting pair of SNPs by calculating the effect of disrupted interaction on the RF prediction error rate [27].

In addition to the good performance of RF method on datasets with epistasis, benchmark studies have recognized the RF classification algorithm to be among the best classifiers for the majority of the real-world datasets [28, 29]. RF is conveniently interpretable with built-in feature importance scores implemented in a majority of popular programming languages and analytics platforms including Python and R. These are calculated with entropy or Gini importance criterion. Despite the utility of RF feature important scores, some studies have reported a bias introduced by the RF feature importance scores when working with categorical, grouped, and varying types of features [30]. An alternative way to estimate feature importances with the RF classifier is by calculating permutation feature importance (PFI) scores. This metric employs an exhaustive permutation concept where features are permuted one at a time and the importance scores expressed via the difference in the ML algorithm’s performance score. While computationally intensive for large feature sets, this approach is advantageous due to its applicability to any machine learning method of any complexity and, therefore, is independent of the characteristics of any given algorithm.

In this study, we aim to improve the interpretability of RF predictions for genetic data in the presence of non-additive interactions by comparing three feature importance metrics: RF’s built-in feature importance coefficients (BIC), mean SHAP values, and PFI coefficients. We use two real-world datasets with previously described non-additive interactions to compare model interpretation using the two different feature importance score metrics. We also compare the metrics using simulated data with varying levels of interaction, imbalance in case/control ratio, and sample size where the ground truth is known.

## Results

### Evaluation of feature importance metrics performance with simulated datasets

An RF classification algorithm with Gini impurity criterion was fitted on all simulated replicates with non-additive interactions (for details see Methods 2.1). For majority of them, 10-fold cross-validated balanced accuracy (unweighted average of the accuracies calculated on a per-class basis) was estimated as 1.0 or very close to 1.0 (Fig. S1). PFI, SHAP, and BIC metrics were estimated for the fitted RF models and further compared to the real feature importances retrieved with the HIBACHI sensitivity analysis (for details see Methods 1.1–1.4 and 2.2). The percentage of successful rank identification per 100 replicates of each experiment was reported in Table 1. For all combinations of factors that were considered in HIBACHI simulations, PFI metric consistently outperformed BIC and SHAP metrics in the ability to determine feature importance order. The most accurate PFI evaluation was produced for the datasets in the category with sample size 1000, two-way Information Gain (IG) and 50% of cases, where the most important feature (F1) was identified precisely in 91% of replicates, F2–90%, and F3–78%. For the sample size 10,000 category, the most accurate estimate by PFI was done for the three-way IG, with 25% of cases where F1 was identified correctly for 89% of replicates, F2 – for 80%, F3 -for 89%, F4 – for 84%. Overall, feature ranks estimated by PFI metric were at least twice more accurate than BIC estimates for nearly all the corresponding experiments. SHAP metric also had a greater success in feature rank estimation as compared to BIC and were equal to PFI for the settings with the imbalanced datasets with two-way IG and population size 1000 however overall showed a greater error than PFI.

**Table 1:** PFI, BIC and SHAP success in identification of feature ranks in datasets with two-way and three-way epistatic interactions. It is expressed as the percentage of a match of a metric rank’s estimate with the true feature rank that was retrieved with the HIBACHI sensitivity analysis

**Sample size 1000**												
**Two-Way IG**							**Three-Way IG**					
**% of cases:**	**25%**			**50%**			**25%**			**50%**		
**Metrics:**	**PFI**	**BIC**	**SHAP**	**PFI**	**BIC**	**SHAP**	**PFI**	**BIC**	**SHAP**	**PFI**	**BIC**	**SHAP**
F1	70%	41%	**71%**	**91%**	42%	82%	**80%**	38%	57%	**79%**	18%	68%
F2	**63%**	41%	62%	**90%**	42%	82%	**69%**	33%	45%	**60%**	36%	52%
F3	**93%**	84%	89%	78%	**82%**	81%	**79%**	33%	53%	71%	18%	**73%**
F4	17%	17%	15%	15%	17%	16%	**74%**	55%	58%	11%	**14%**	11%
F5	5%	4%	5%	4%	5%	3%	33%	31%	29%	5%	6%	4%
**Sample size 10,000**												
**Two-Way IG**							**Three-Way IG**					
**% of cases:**	**25%**			**50%**			**25%**			**50%**		
**Metrics:**	**PFI**	**BIC**	**SHAP**	**PFI**	**BIC**	**SHAP**	**PFI**	**BIC**	**SHAP**	**PFI**	**BIC**	**SHAP**
F1	**79%**	36%	73%	**83%**	39%	75%	**89%**	42%	68%	**86%**	19%	76%
F2	**79%**	32%	73%	**83%**	39%	75%	**80%**	34%	49%	**75%**	34%	56%
F3	**100%**	87%	99%	**81%**	79%	81%	**89%**	33%	66%	**85%**	22%	70%
F4	13%	12%	13%	44%	38%	40%	**84%**	62%	69%	**53%**	51%	52%
F5	5%	4%	5%	15%	13%	14%	40%	38%	37%	11%	9%	10%

F1, F2, etc. – feature ranks, *PFI* permutation feature importance, *BIC* build-in coefficients, *SHAP* shapley additive explanations, *IG* information gain

**Fig. 1:**
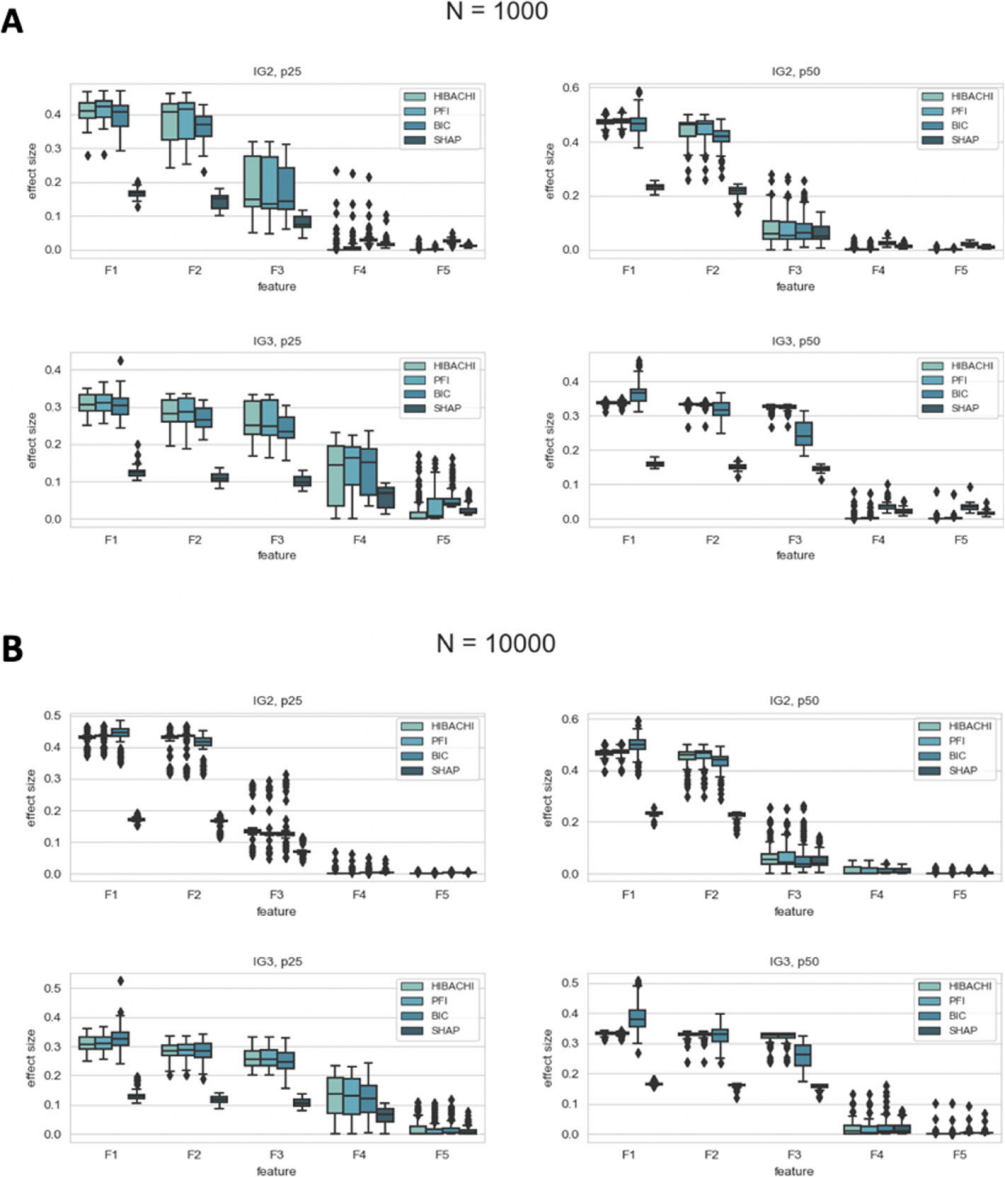
Effect size per feature rank estimated by PFI, BIC, SHAP and HIBACHI sensitivity analysis for sample size 1000 (**a**) and 10,000 (**b**). *F1, F2,* etc. *– feature ranks, PFI -permutation feature importance, BIC – build-in coefficients, SHAP - shapley additive explanations, IG- Information Gain, p25, p50 – percentage of cases*

**Fig. 2:**
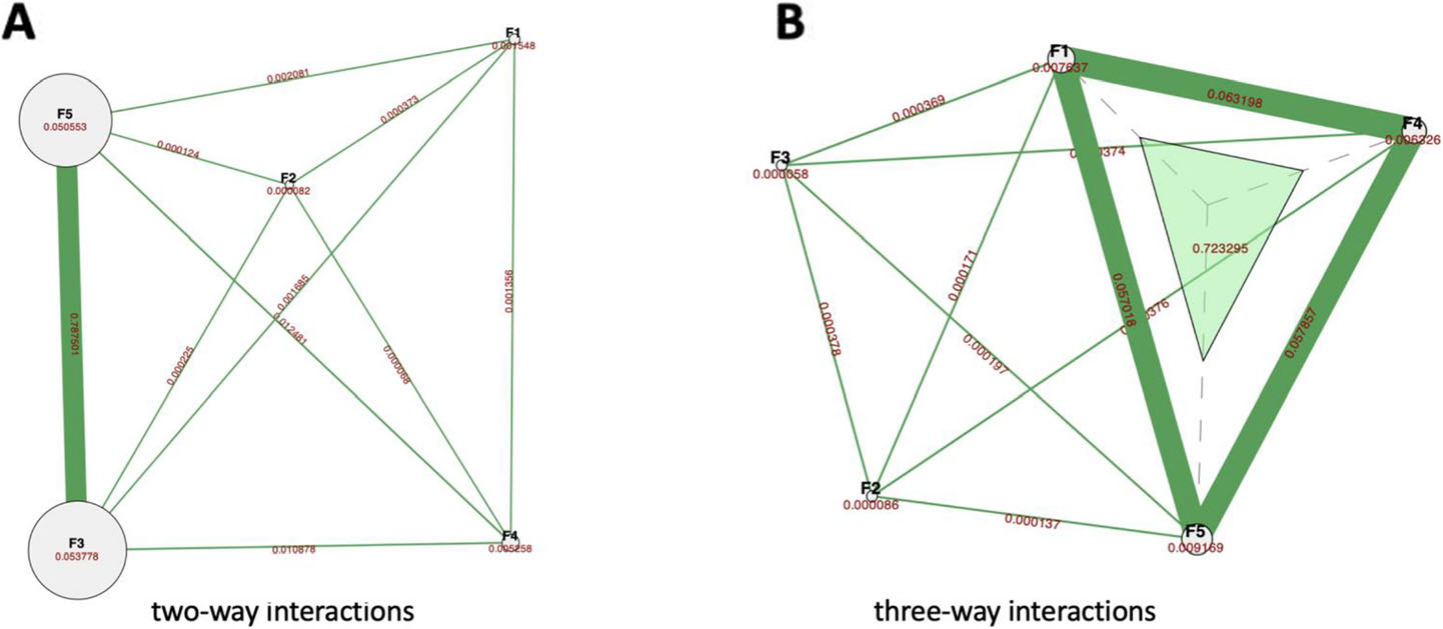
Example of ViSEN plots for the datasets with two-way (**a**) and three-way (**b**) interactions. Features main effects, two-way and three-way IG values are noted respectively

Interestingly, for the majority of replicates of the experiments with the two-way IG, the PFI metric was able to identify the top three features, while for the three-way IG this came up to the top four features. We investigated further into the population of the feature importance effect sizes and discovered that experiments with the two-way interactions observe the same range of effect sizes for the top two important features, while the effect sizes of the bottom two features are 2–3 orders of magnitude smaller than the top ones or zero (Fig. 1a, b). To support this observation Table 2 report the percentage of replicates with zero effect size at each feature importance rank: for the majority of the experiments with the two-way IG, two out of five features didn’t have an effect on the phenotype. A more thorough exploration of the fitness function landscape confirmed that in the majority of cases only one pair or trio of features had enough time to evolve strong non-additive interactions due to the limited simulation time. Hence, we observe that the interacting pair of features share the major informative contribution towards the phenotype, while non-interacting features contribution effect is orders of magnitude smaller and insufficient to be detected accurately by both importance metrics (Fig. 2a, b).

**Table 2:** Percentage of the features with zero effect size for every rank position

**Sample size 1000**				
**Information gain:**	**IG2**		**IG3**	
**Percent of cases:**	**25%**	**50%**	**25**	**50%**
F1	0%	0%	0%	0%
F2	0%	0%	0%	0%
F3	0%	15%	0%	0%
F4	76%	70%	23%	83%
F5	91%	92%	65%	92%
**Sample size 10,000**				
**Information gain:**	**IG2**		**IG3**	
**Percent of cases:**	**25%**	**50%**	**25%**	**50%**
F1	0%	0%	0%	0%
F2	0%	0%	0%	0%
F3	0%	17%	0%	0%
F4	85%	54%	16%	47%
F5	94%	84%	59%	88%

F1, F2, etc. – feature ranks, *PFI* permutation feature importance, *BIC* build-in coefficients, *SHAP* shapley additive explanations, *IG* information gain

### Evaluation of the feature importances in real-world datasets with non-additive interactions

**Fig. 3:**
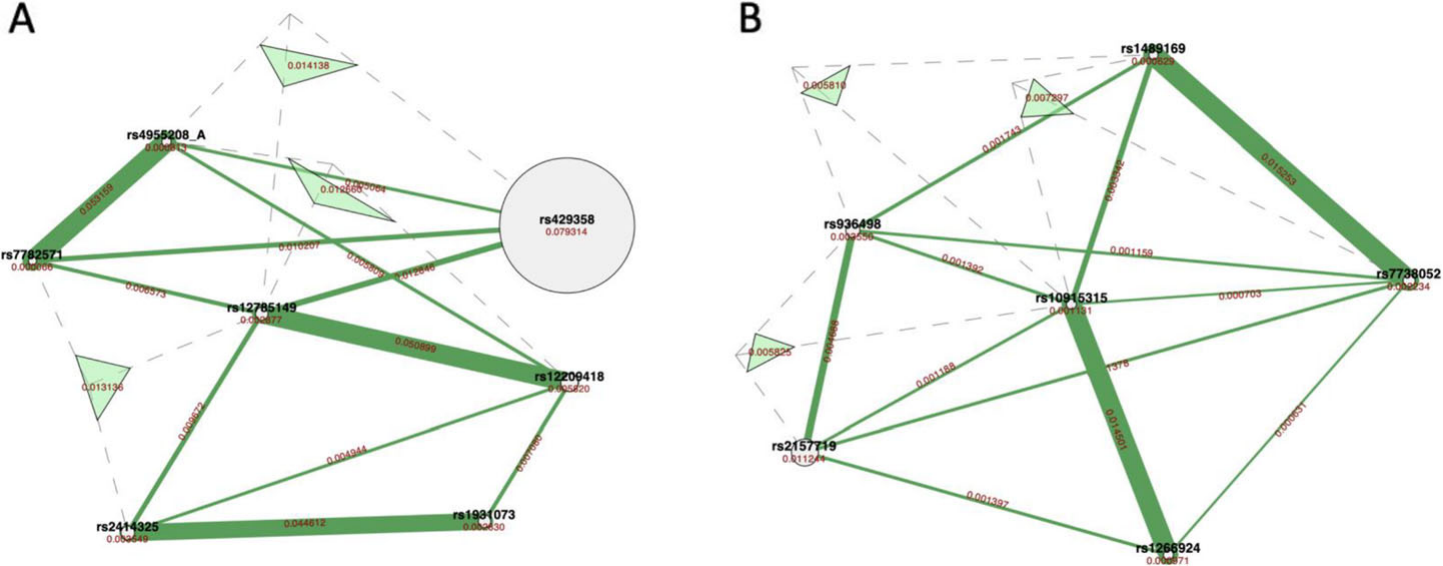
ViSEN plots for selected SNPs in Alzheimer’s (**a**) and Glaucoma (**b**) datasets. SNPs main effects, two-way and three-way IG values are noted respectively

**Fig. 4:**
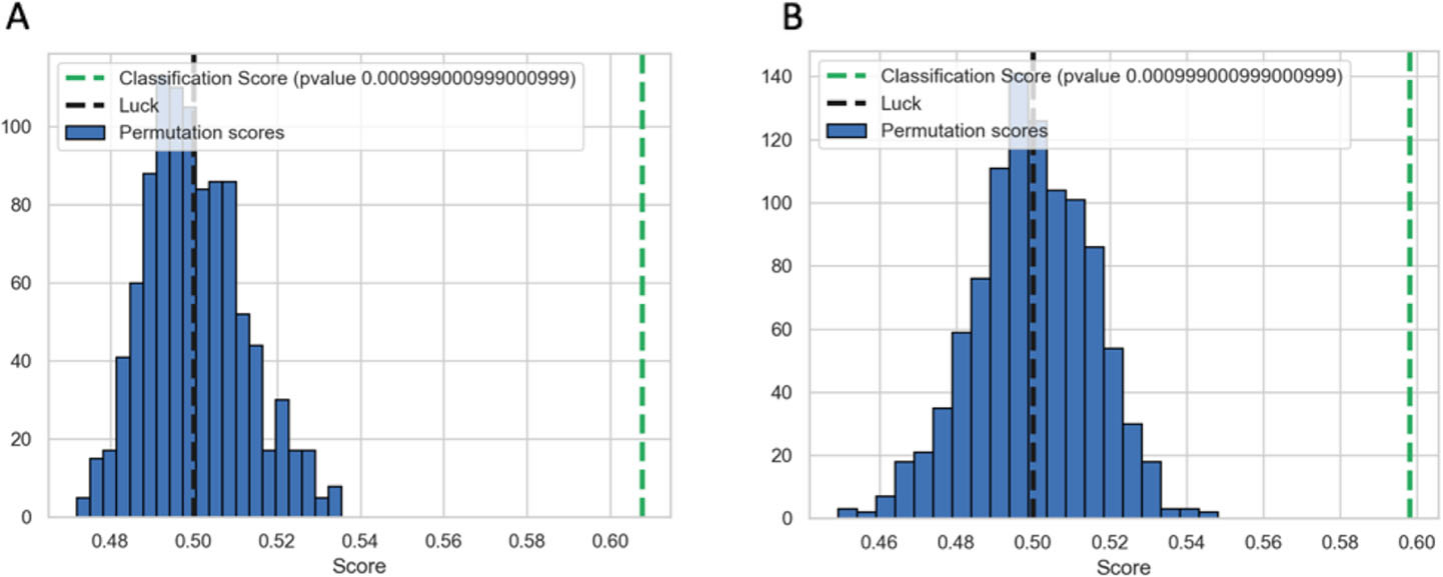
Permutation test scores for the RF classifier for Alzheimer’s (**a**) and Glaucoma (**b**) datasets

**Fig. 5:**
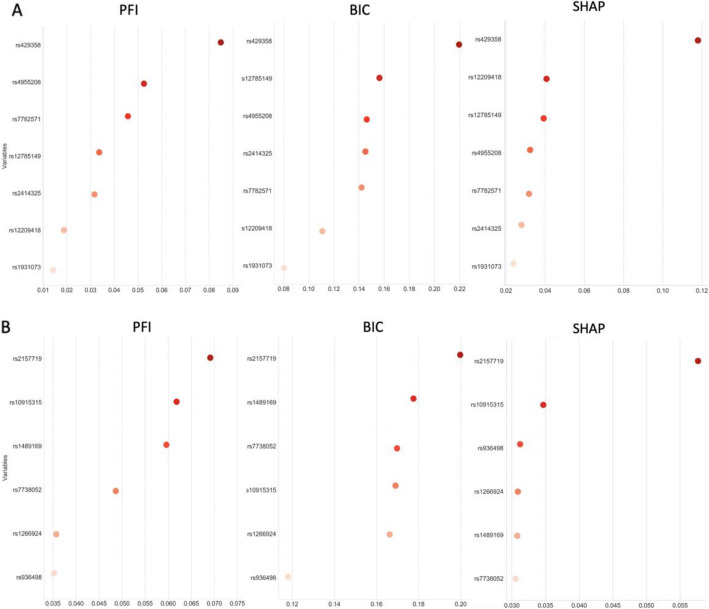
PFI and BIC estimates for Alzheimer’s (**a**) and Glaucoma (**b**) datasets. *PFI -permutation feature importance, BIC – build-in coefficients, SHAP - shapley additive explanations*

We used the HIBACHI simulation framework to obtain datasets with strong non-additive interaction between the features - genetic variants and the phenotype. We were able to demonstrate that regardless of the dataset’s parameters, the best method to determine features’ informative contribution to the phenotype is PFI. To validate our findings for real-world data we analyzed two datasets with complex disease phenotype which previously have been identified to have non-additive interactions among SNPs. The subset of seven SNPs was selected from the Alzheimer’s disease data and the subset of six SNPs was selected from the Glaucoma dataset as described above (see Methods 2.4). A ViSEN analysis and plot for the Alzheimer’s dataset (Fig. 3a) revealed one SNP with large independent effect affiliated with ApoE gene (rs429358, MI 0.08) – a known risk factor for Alzheimer disease, and three strong two-way gene-gene interactions (rs4955208 - rs7782571, IG 0.05; rs12785149 - rs12209418, IG 0.05; rs2414325 - rs1931073, IG 0.05). ViSEN analysis of the Glaucoma dataset revealed two strong gene-gene interactions (rs7738052 - rs1489169, IG 0.015, and rs10915315 - or rs1266924, IG 0.015). We built a predictive model for each dataset with the RF classifier: the model was tuned with grid search and had classification balanced accuracy 62.6% for the Alzheimer dataset, and 60% for the Glaucoma dataset. We verified the significance of the cross-validated balanced accuracy scores of the optimized classifiers by doing a permutation test [31] and confirmed that both models have reliable classification performance (Fig. 4). We further calculated PFI, BIC and SHAP estimates for the optimized RF models. For Alzheimer dataset (Fig. 5a) the most important feature, according to PFI, BIC and SHAP metrics, is the SNP with the largest independent effect – rs429358. This SNP has been discovered by ViSEN analysis and expected to have strong effect on the phenotype. However, the consecutive feature importance order diverged between two metrics. According to PFI rank, SNPs located at second and third position belong to the same interaction and valued with the highest IG out of all calculated pairwise interactions for this dataset (Fig. 3a). At the same time, SNPs located at the second and third position by BIC ranking didn’t create a strong pairwise interactions with each other. SHAP metric has placed SNPs that belong to the second strongest pairwise interaction at the second and third position correspondingly.

We observed a similar discrepancy between the metric rank evaluations for the Glaucoma datasets (Fig. 5b): while SNP rs2157719 has the largest main effect among all SNPs and was assigned to be the top feature by all compared metrics, the following order of SNPs was outlined conversely by different metrics. SNP rs10915315 has the largest number of detected pairwise interaction among all SNPs and is ranked second by both PFI and SHAP, while only fourth by BIC; SNP rs1489169 has three pairwise interactions (including the strongest one) detected and is ranked third by PFI and second by BIC. Interestingly, both metrics identified the less important and non-important SNPs in the same order, suggesting that the issue with the discrepancy between the metrics evaluations should be address for the top features only.

### Evaluation of the two-way and three-way permutation feature importance metrics for interaction detection

We further investigated whether two- and three-way PFI could be used in the interaction detection and we evaluated the success of these metrics for the HIBACHI simulated datasets. We calculated the IG of all pairs and trios of features for each dataset and evaluated the detection success of the strongest interaction with the top two-way and three-way PFI correspondingly (Table S1). Additionally, we calculated the interaction detection success with the top two and three features identified by single feature PFI (Table S1). As it can be seen from the table, a single feature PFI metric has a substantially higher success in interaction detection across all experimental settings. We also observed a clear trend for decreased success rate for the experiments with the imbalanced datasets (only 63 and 54% for the datasets with the two-way interactions and 48 and 53% for the datasets with the three-way interactions). We suggest that this trend can be observed due to the presence of the second and third interaction pair and/or trio since HIBACHI fitness function is set up to maximize all possible interactions combinations. Moreover, it is possible that imbalanced dataset creates an additional selective pressure for the second and third interactions to appear during the GP process. This possibility is supported by increased effect size of the third most important feature in the IG2 datasets (F3) and the fourth most important feature (F4) in the IG3 datasets with the case/control imbalance parameter (p25) as compared to balanced experiments (p50) (Fig. 1).

We calculated IG values for all pairs and trios of features in all HIBACHI datasets and visualized the distribution of the top three strongest interactions stratified by the status of its detection success by two-way and three-way PFI (Figure S2). In the majority of settings, the effect size of the second and third interaction was higher for the datasets where two-way and three-way PFI failed to detect the strongest interaction. Therefore, we suggest using single feature PFI as more precise metric for non-additive interactions detection, especially for complex datasets where the presence of more than one interaction is expected.

## Discussion

In this study we addressed the problem of interpretability of the RF classification method predictions in datasets with non-additive interactions by comparing the power of various feature importances metrics: PFI, SHAP and BIC. The real-world data analysis which included genetic datasets with Alzheimer’s and Glaucoma patients confirmed the discrepancy in feature importance ranking among the studied metrics. We analyzed the metrics’ performances for HIBACHI-simulated datasets with a range of parameters including sample size, case/control imbalance and degree of interaction complexity and find the PFI metric has superior performance. We further analyzed the ability of single- and multi-feature PFI metrics to identify the strongest non-additive interaction and the single-feature PFI consecutive ranking appeared to be the most accurate interaction detection approach.

Multiple research studies suggested that epistatic interactions are widespread by nature and that many genes work in an interactive manner [5]. Epistatic interactions are expected to be found in a variety of pathophysiological processes with one of them most likely to be Alzheimer’s disease [8]. The most powerful predictor of Alzheimer’s disease at this time is ApoE E4 gene variation: one or two copies of ApoE is associated with an increased risk of disease onset [32]. However, some carriers of ApoE E4 variation haven’t developed an Alzheimer’s disease so it is very likely that other genetic factors are involved in disease’s pathophysiology. ViSEN entropy-based analysis revealed several strong pairwise genetic interactions, along with the known largest independent signal from the ApoE variant (rs429358) (Fig. 3a). Furthermore, ViSEN method allocated non-additive interactions within the Glaucoma disease dataset: several strong pairwise interactions in addition to the independent main effect contribution from the SNP affiliated with retinal ganglion cells pathology (rs2157719) have been confirmed (Fig. 3b). Evaluation of an informative contribution of a single genetic factor involved in the two- or three-way interactions towards the phenotypic outcome is a challenging analytical task and it requires a non-linear solution which RF classifier is notorious for. Therefore, we aimed to identify whether RF classifier is capable to detect genetic signatures that were previously confirmed by ViSEN analysis and whether different RF’s feature importance metrics will be able to agree on the rank.

Three feature importance metrics were considered, PFI, BIC and SHAP, and each was compared after RF analysis of data derived from genome-wide association studies of Glaucoma and Alzheimer’s. The resulting feature ranking confirms the lack of consensus between the studied metrics (Fig. 4). Indeed, while all three metrics identified the SNPs with the largest independent main effects (rs429358 and rs2157719) as a top feature in both real-world datasets, genes involved in the non-additive interactions have been assigned different importance ranks by different metrics. More specifically, in the Alzheimer data, the second and the third most important feature predicted by PFI and SHAP belong to the same interacting pairs (rs4955208 and rs7782571, and rs12209418 and rs12785149), while the same rank positions predicted by BIC were occupied by SNPs that do not belong to the same genetic interaction (Fig. 3a, Fig. 5a). A similar discrepancy has been observed in the Glaucoma dataset: PFI, BIC and SHAP indicated different interacting pairs of SNPs as the second and third most important feature (Fig. 5a), however SNP predicted to be the second most important by PFI and SHAP is a part of four additional SNP-SNP interactions, while SNP predicted second by BIC is a part of only three interacting SNP pairs (Fig. 3b).

**Fig. 6:**
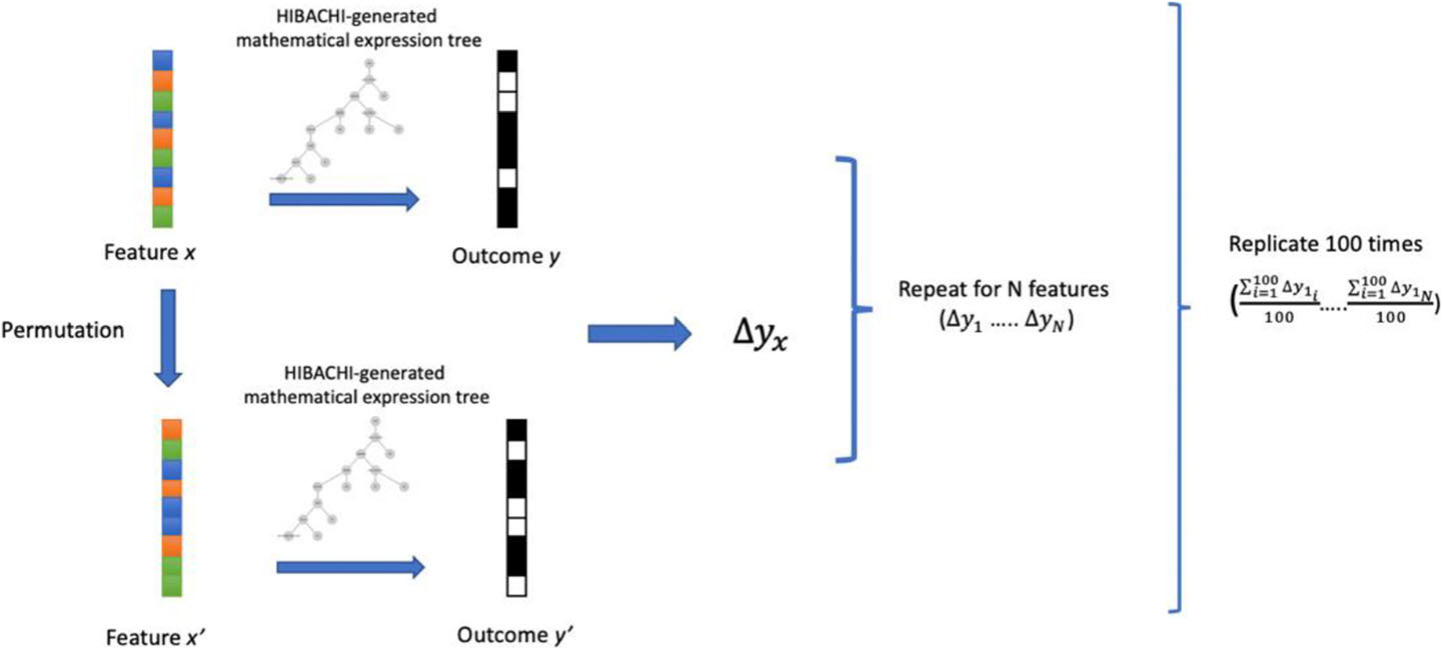
Scheme of sensitivity analysis for the HIBACHI experiments

Such uncertainty has been associated with RF predictions in the past and we attempted to reveal the true interpretation with the computational experiments driven by HIBACHI simulations. The HIBACHI framework has the ability to consider any desirable biological concept in the form of mathematical expressions that define the genotype-phenotype relationship and evolve models that can be used to simulate data consistent with that relationship. We set up a simulation goal to maximize two- or three-way interactions among features and compared RF’s feature importance metrics with the sensitivity analysis results of the simulated data that provided us with the ground truth information about the feature ranks (Fig. 6). In all HIBACHI experimental setups, which included such factors as the proportion of cases and controls, sample size and interaction complexity, PFI metrics produced the most precise feature ranking (Table 1, Fig. 1). Although BIC and SHAP metrics misplaced feature ranks for the large percentage of replicates with BIC failed to identify the majority of them, it correctly identified features that belong to the interactive pair or trio by putting them as a top-ranked two and three features correspondingly. Therefore, we can suggest that while BIC and SHAP metrics can still be useful, when there is a need for an absolute precision, PFI estimation method should be used.

We further aimed to establish whether a two-way and three-way PFI could be used as a metric for non-additive interaction detection in the simulated datasets. Interaction detection success with the multi-feature PFI underperformed in all experimental settings and our investigation identified that this is most likely due to the presence of an additional non-additive interactions of smaller strength. Indeed, the HIBACHI datasets evolved to have multiple non-additive interactions of different strength in addition to the strongest one and this was especially prevalent in settings with the imbalanced case/control ratio. Permutating one feature which is involved in the non-additive interaction would remove its informative effect in full and, therefore, the combined permutation effect of features from the different non-overlapping interactions (even orders of magnitude weaker) would be picked up as the top feature combination by multi-feature PFI metric. Therefore, a single-feature PFI metric provides a more accurate non-additive interaction detection with its effect size distribution stratification in complex data which are most likely very common in the genetics of human diseases. This, however, may not be true for all real-world scenario, for example, if two and more interactions of equal strength are present, a single-feature PFI will not be useful and a better method is needed for this aim.

PFI has some limitations we didn’t cover in our experiments – it is particularly sensitive to highly correlated features. In case such features are present in the dataset, PFI requires a special preprocessing directed onto the removal of such features. An example of this could be a hierarchical clustering based on the Spearman’s rank correlation coefficients with following cluster-based filtering. In future studies, other feature importance techniques may be considered which can include joint importance by maximal subtrees, joint variable importance and corrected Gini importance score [33] as well as a more complex epistatic schemes could be explored (e.g. different combinations of marginal and interaction effects as in [34], multiple strong interactions and/or combination of two- and three-way interactions). Within our simulation framework we mostly observed one or two strong interactions and this might be an overly simplistic given the complexity of common diseases such as Alzheimer’s and glaucoma.

## Conclusion

In this study, we performed a comparative analysis of feature importance metrics with the aim to improve Random Forest’s interpretability in datasets with complex interactions. By analyzing both real and simulated data, we established that the permutation feature importance metric provides more precise feature importance rank estimation in the presence of non-additive interactions.

## Methods

### Random forest and its properties

RF is a popular ML algorithm because it often demonstrates good performance while remaining relatively easy to optimize and interpret. RF algorithms belong to a Bagging (Bootstrap Aggregation) type of ensemble ML methods where a group of weak learners in a form of decision trees (DT) classify the outcome using majority vote. Decision trees are sensitive to the data they are trained and often suffer from high variance problem especially when the depth of the tree is high. To address that RF algorithm trains each tree on a subset of samples drawn from the complete dataset with the bootstrap procedure. An additional source of randomness within the RF algorithm is introduced during the construction of a tree when selecting a split node from the random sample of features (in place of the greedy search through all feature set like in DT). These two sources of randomness aim to decorrelate weak learners and correspondingly decrease the variance of an estimator by combining diverse trees prediction via majority vote.

During the construction of a DT, the decrease in the error function can be calculated for a feature at each split node. In a classification task, this is often done via estimating the Gini score or the entropy score. The function decreases can be averaged across all trees and returned as feature importances score (the greater the decrease the higher feature importance). Feature importance scores are often conveniently implemented as a RF function which makes this method more interpretable.

### Permutation feature importances

**Fig. 7:**
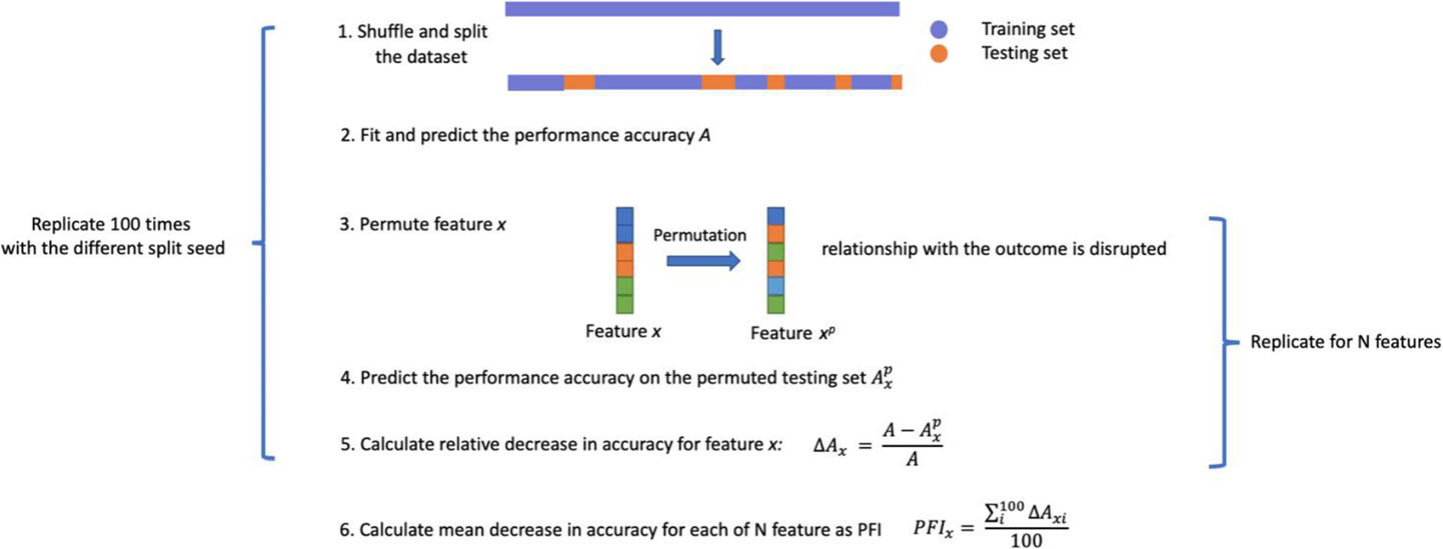
Scheme of the permutation feature importance custom implementation

Permutation feature importance metrics were first introduced by Breiman in his Random Forest manuscript [35] and further extended by Altmann [36] to correct for the bias of the RF’s Gini importance and entropy criterion for feature selection. We utilize a custom implementation of PFI which could be applied to any machine learning classification and regression algorithms (Fig. 7). Here, PFI metric is calculated with following steps: 1) the dataset is shuffled and split into the training and testing datasets 2) the model is fitted on the training dataset and the balanced accuracy is estimated on the testing dataset, 3) feature 1 out of N is permuted for the testing dataset 4) the balanced accuracy is estimated on the permuted testing dataset 5) the relative decrease of the permuted and non-permuted balanced accuracies is calculated and stored as relative decrease in accuracy, 4) step 2 and 3 are repeated for the remaining N-1 features, 5) steps 1 through 4 are replicated for N-1 times with the new seeds for shuffle and split procedure, 6) mean of relative decrease in accuracy per feature is calculated across the splits and is used as features’ PFI value. Retrieved PFI values were normalized to sum to 1.

### Two-way and three-way permutation feature importances

To calculate two- and three-way PFI we used the methodology described above with two or three features permuted at step 3 correspondingly.

### SHAP values

SHAP (SHapley Additive exPlanations) method [4] uses a cooperative game theory approach to explain the machine learning model predictions with SHAP values which are calculated as a weighted average of features’ marginal contribution. SHAP is commonly used as a local explanation tool, however it also provides the approximation for a global solution via mean SHAP values metric and we will be using this as an alternative metric for Random Forest feature importances success comparison.

## Evaluation of the feature importance metrics

### Data simulation using HIBACHI

We used Heuristic Identification of Biological Architectures for simulating Complex Hierarchical Interactions (HIBACHI) software to simulate genetic datasets with non-additive epistatic interactions of different complexity. The HIBACHI method employs biological and mathematical frameworks to connect genotype and phenotype [37] At the bottom of the biological framework is the concept of information transfer from the DNA sequence to a clinical phenotype through complex interactions at multiple levels: gene expression, pathway, and cell. Within this framework, a population of samples is expressed as a collection of genes (genotype) each of which has three variants 0, 1 or 2. The mathematical framework’s goal is to specify a relationship between genotype and phenotype in terms of logical, arithmetical and other functions. HIBACHI merges the frameworks by evolving a mathematical expression tree which when applied to a genotype, generates a binary clinical phenotype.

HIBACHI employs Genetic Programming (GP) as an optimization engine. One of the key characteristics of GP is a fitness function which is represented through the mathematical expression that satisfy a specific objective of interest defined by user. This objective could be a performance of a machine learning pipeline, complexity of genetic interactions, odds ratio of genetic effect sizes, length of expression tree, etc. or a combination of those in the form of the multi-objective fitness function. Additionally, user to allowed to specify the length of the optimization (by specifying the number of generations), the population size (number of samples) and genotype size (number of genes) of the dataset.

At the beginning of the GP optimization process, a population of individuals with random mathematical expression trees is initialized and is further subjected to mutational and recombinational processes. This process serves as a source of variation for the expression trees and have a pre-defined rate. After that, a user-defined fitness function is calculated and the best-fitted individuals are selected for the next round. At the last optimization round, an overall best-fitted individual is used as an output dataset.

For the aims established within this study, we wanted to generate datasets with non-additive epistatic interactions that would involve two and three genetic variants and, therefore, we have defined a fitness function that maximizes two-way and three-way information gain term (indicate the synergy or the redundancy between two/three variables (or genotype) with respect to the third/fourth variable (or phenotype)). Entropy-based IG approach to detect epistatic interactions has been introduced by Moore et al. [38]. Two-way IG defined as:

$$ IG\left(X;Y;Z\right)=I\left(X,Y;Z\right)-I\left(X;Z\right)-I\left(Y;Z\right), $$

Where *I* is mutual information that describes the dependency between variables X, Y and Z. It measures the reduction of uncertainty of one variable (Z) given the knowledge of others (X and Y) It is expressed with entropy terms:

*I*(*X*, *Y*; *Z*) = *H*(*Z*) − *H*(*Z*| *X*, *Y*); *I*(*X*; *Z*) = *H*(*Z*) − *H*(*Z*| *X*); *I*(*Y*; *Z*) = *H*(*Z*) − *H*(*Z*| *Y*),

Where entropy *H* defined with the probability mass function:

$$ H\left(Z|X\right)=H\left(Z,X\right)-H(Z);H(Z)=-\sum \limits_{z\in Z}p(z)\log p(z); $$

$$ H\left(Z,X\right)=-\sum \limits_{z\epsilon Z}\sum \limits_{x\epsilon X}p\left(z,x\right)\log p\left(z,x\right) $$

Definitions of three-way IG can be found in Hu et al., 2013 [39]. We used the following version of this term:

$$ IG\left(X;Y;Z;N\right)=I\left(X,Y,Z;N\right)- IG\left(X;Y;N\right)- IG\left(X;Z;N\right)- IG\left(Y;Z;N\right)-I\left(X;N\right)-I\left(Y;N\right)-I\left(Z;N\right) $$

Additionally, a second fitness objective was set up as maximization of expression tree length, to encourage multiple combinations of genetic interactions. In addition to the variability in the interaction complexity, the following factors have been considered in the HIBACHI experimental schemes: percent of cases (25 and 50%) to address an imbalanced dataset structure, and sample size (1000 and 10,000). Each experimental setup was reproduced 100 times using random seed generator and the whole population of replicates was considered in the consecutive analysis. All simulated data used here is available upon request.

### Sensitivity analysis

We implemented a HIBACHI-based sensitivity analysis to determine the true feature importances ranks and the effect sizes. For this the permutation-based framework was implemented with the following steps (Fig. 6): 1) split HIBACHI-generated dataset into the outcome vector and the feature set 2) permute feature vector 1 out of N and re-evaluate the outcome by applying the HIBACHI-generated expression tree 3) calculate the dissimilarity of the outcome as of mismatch between the perturbed and unperturbed feature set 4) repeat the estimate 2) for the remaining feature and normalize the counts by the total sum; 5) replicate steps 2–4100 times and calculate the average of the replicates per feature as a final true feature importance score. Sensitivity scores were further normalized to sum to 1.

## Real world data analysis

To examine the convergence of the RF’s feature importance metrics we used two real-world datasets with evidence for non-additive interactions. The first includes preselected SNPs from a genome-wide association study of Alzheimer’s Disease while the second includes preselected SNPs from a genome-wide association study of Primary Open Angle Glaucoma (POAG). The Alzheimer’s dataset came from the Alzheimer’s Disease Neuroimaging Initiative during which functional MRI was taken every 6 to 12 months for patients with three health conditions (neuro-typical (identified here as controls), and mild cognitive impairment and Alzheimer’s disease (identified here as cases)). A computational evolution system [40] identified a model of seven SNPs with evidence of non-additive interactions and a classification accuracy of 0.738. Among these SNPs are the SNP with the large main effect that is located in the APOE gene (rs429358) – a known risk factor for Alzheimer disease, four SNPs located within genes with known functionality/disease state (rs1931073 – an intergenic region near the PPAP2B gene that is participating in cell-cell interactions, rs7782571 – near the ISPD gene that is associated with the Walker-Walburg syndrome, rs4955208 – in the OSBPL10 genes which are expressed into intracellular lipid receptor, rs12209418 – in the PKIB gene that codes a protein kinase inhibitor) and two remaining SNPs (rs2414325, rs12785149) are located within genes with unknown functionality.

The glaucoma dataset came from the Glaucoma Gene Environment Initiative study and contained with POAG individuals identified as cases and healthy individuals as controls. This dataset has been previously analyzed by Moore at al [41]. with the EMERGENT algorithm that resulted in the identification of a model of six SNP’s with evidence of non-additive interactions and a classification accuracy of 0.615. Two of these SNPs (rs2157719, and rs1266924) are located within the genes that were previously associated with glaucoma disease, two SNPs (rs10915315, and rs1489169) are located within the genes that are associated with glaucoma-non-related diseases and relevant pathology, and two more SNPs (rs936498, and rs7738052) are located within the genes that were not previously associated with any disease, but have a known functionality that is relevant to visual cortex and retina development.

We used the visualization of the statistical interaction network (ViSEN) method [42] to analyze and visualize SNP main effects, and two-way and three-way gene-gene interactions among SNPs for real-world datasets. The ViSEN method calculates the mutual information (MI) between individual SNP (genotype) and the phenotype, the pairwise interaction between every pair of SNPs and the phenotype and the three-way interaction between every combination of three SNPs and the phenotype via the IG term. A positive IG indicates synergistic (i.e. non-additive) effects of SNPs on the phenotype. The IG metrics used in the ViSEN method were designed to detect pure epistatic interactions and excluded all lower-order effects (by subtracting all main effects and pairwise synergies in cases of the three-way term).

## Supplementary Information

**Additional file 1: Figure S1.** Distribution of balanced accuracy of Random Forest prediction for population of HIBACHI experiments. Experiments are indicated with population size (1000 or 10000), interaction complexity (IG2 or IG3) and proportion of cases and controls (p25 or p50).

**Additional file 2: Figure S2.** Effect size of the top three interactions stratified by the multi-feature PFI detection success status.

**Additional file 3: Table S1.** Interaction detection success for multi-feature PFI and single feature PFI.

**Additional file 4: Table S2.** Spearman correlation coefficient for all features (A) and for the top 3 features (B).

## Data Availability

The datasets used and/or analyzed during the current study are available from the corresponding author on request.

## Abbreviations

RF: Random Forest
PFI: Permutation feature importance
HIBACHI: Heuristic Identification of Biological Architectures for simulating Complex Hierarchical Interactions
BIC: Build-in coefficients
