# Supplementary material for: A comparison of methods for interpreting random forest models of genetic association in the presence of non-additive interactions
Source: BioData Min. 2021 Jan 29;14:9. doi: 10.1186/s13040-021-00243-0 (PMC7847145; doi:10.1186/s13040-021-00243-0)

Figure S1. Distribution of balanced accuracy of Random Forest prediction for population of HIBACHI experiments. Experiments are indicated with population size (1000 or 10000), interaction complexity (IG2 or IG3) and proportion of cases and controls (p25 or p50)


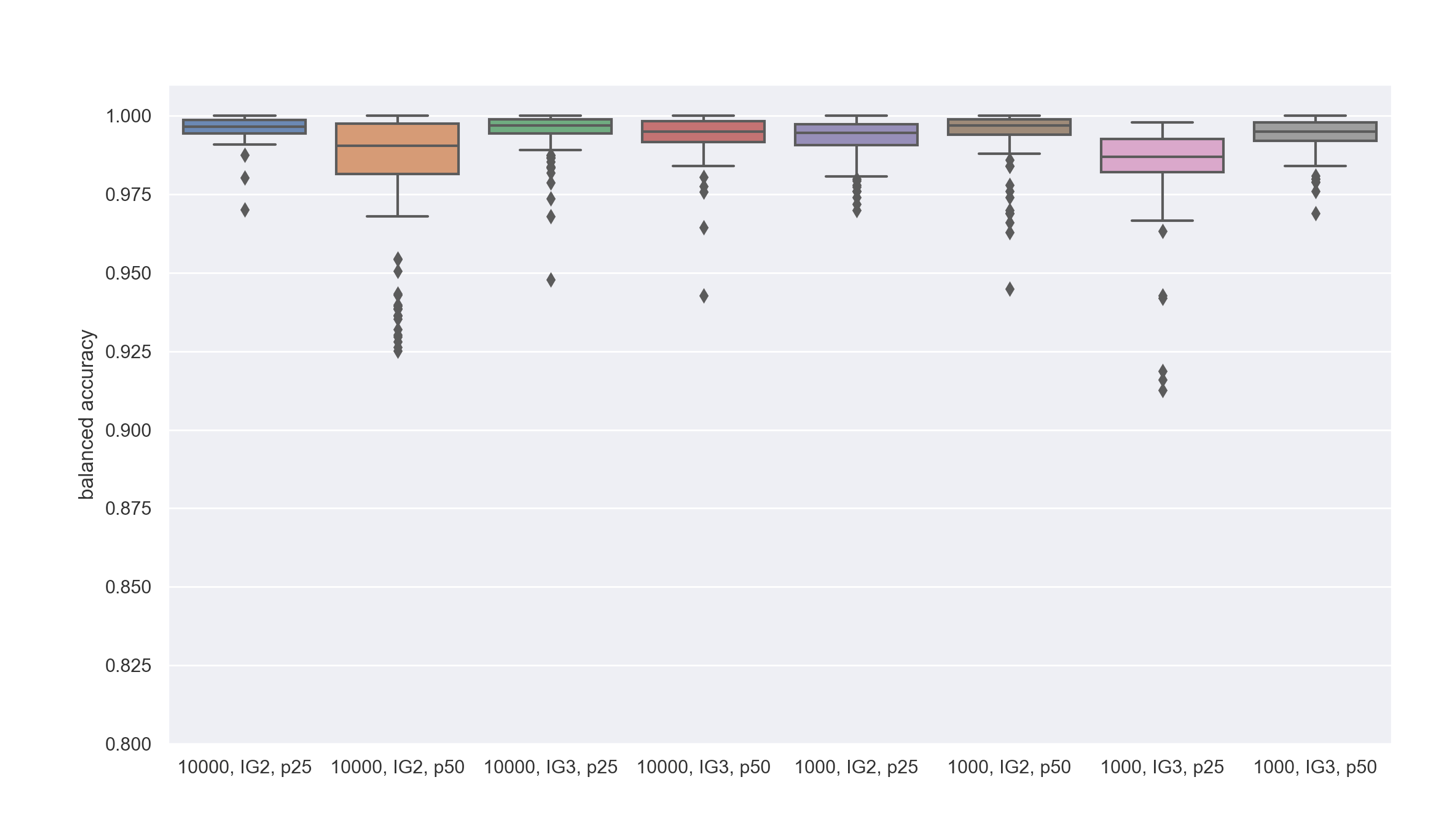

Supplement: Supplementary file 1 — Additional file 1: Figure S1. Distribution of balanced accuracy of Random Forest prediction for population of HIBACHI experiments. Experiments are indicated with population size (1000 or 10000), interaction complexity (IG2 or IG3) and proportion of cases and controls (p25 or p50). [file 13040_2021_243_MOESM1_ESM.docx]
