# Supplementary material for: A comparison of methods for interpreting random forest models of genetic association in the presence of non-additive interactions
Source: BioData Min. 2021 Jan 29;14:9. doi: 10.1186/s13040-021-00243-0 (PMC7847145; doi:10.1186/s13040-021-00243-0)

Figure S2. Effect size of the top three interactions stratified by the multi-feature PFI detection success status

*IG- Information Gain, p25, p50 – percentage of cases, 1000, 10000 – population size*


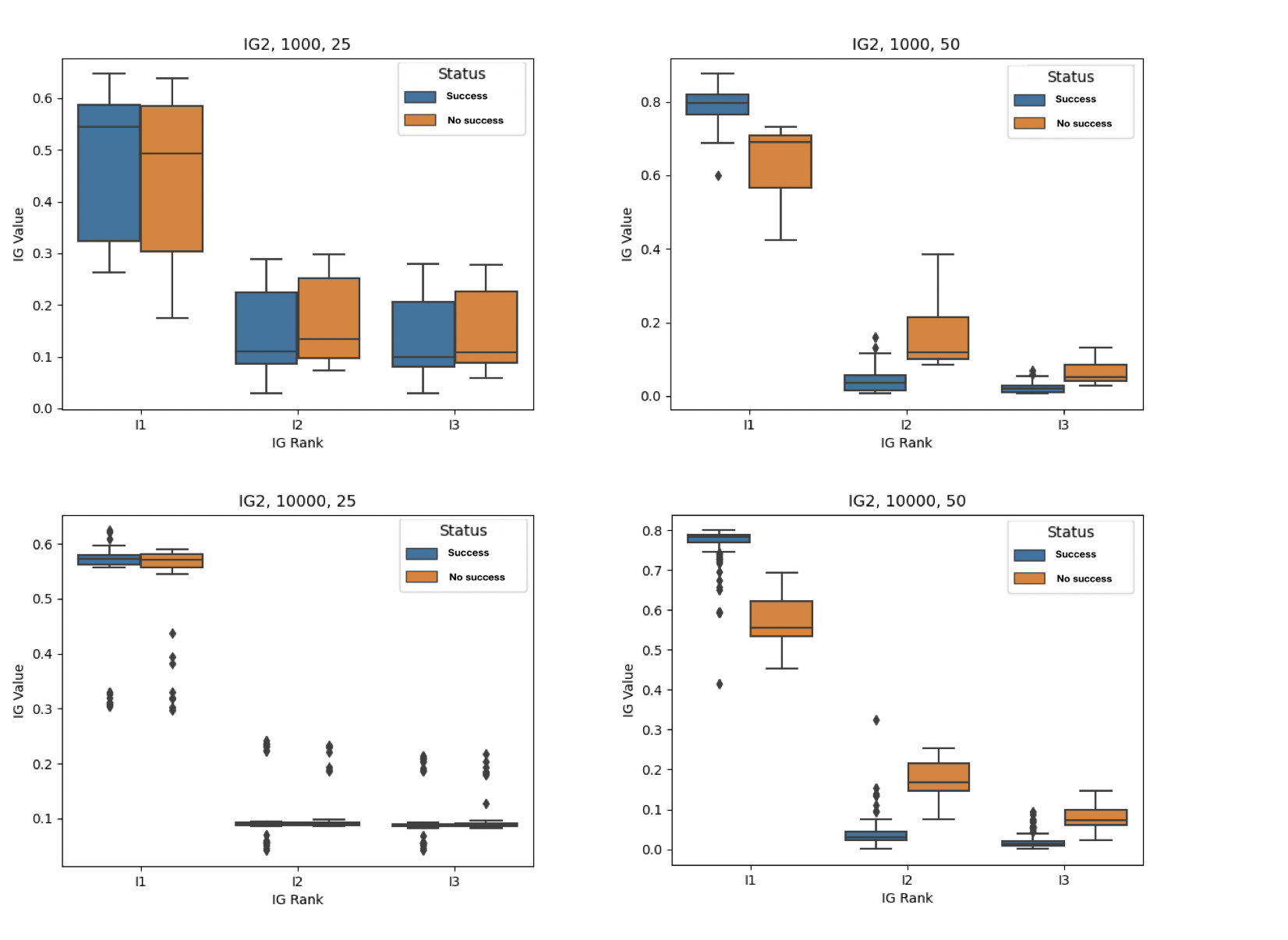

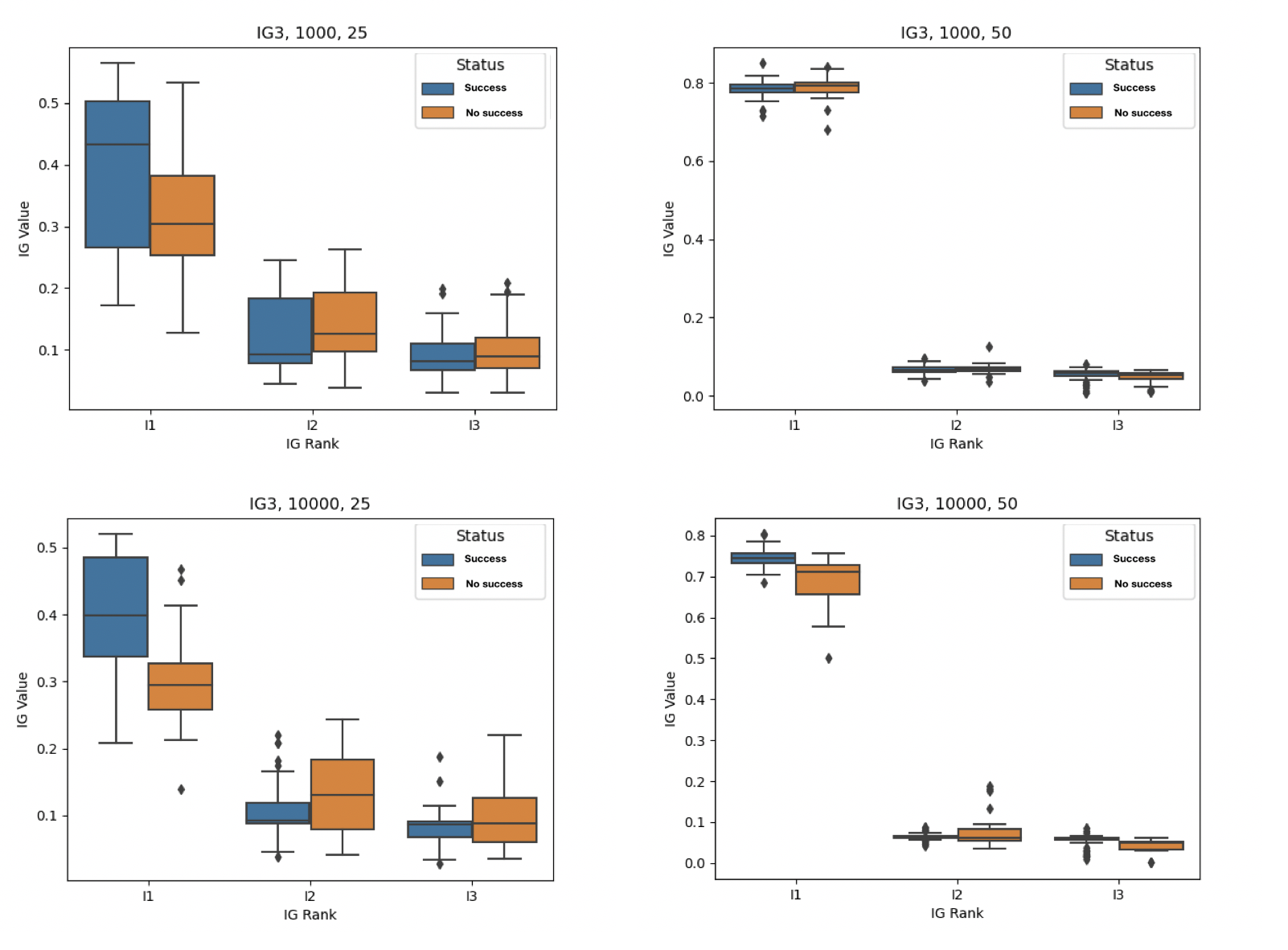

Supplement: Supplementary file 2 — Additional file 2: Figure S2. Effect size of the top three interactions stratified by the multi-feature PFI detection success status. [file 13040_2021_243_MOESM2_ESM.docx]
