# Supplementary material for: A comparison of methods for interpreting random forest models of genetic association in the presence of non-additive interactions
Source: BioData Min. 2021 Jan 29;14:9. doi: 10.1186/s13040-021-00243-0 (PMC7847145; doi:10.1186/s13040-021-00243-0)
Supplement: Supplementary file 3 — Additional file 3: Table S1. Interaction detection success for multi-feature PFI and single feature PFI. [file 13040_2021_243_MOESM3_ESM.docx]

Table S1. Interaction detection success for multi-feature PFI and single feature PFI

*PFI -permutation feature importance, IG- Information Gain, p25, p50 – percentage of cases, N – population size*

| **2-way interaction, detection success %** | | |
| --- | --- | --- |
| **Experiment** | **PFI (top two features)** | **Two-way PFI** |
| IG2, N1000, p25 | 92 | 63 |
| IG2, N1000, p50 | 100 | 75 |
| IG2, N10000, p25 | 100 | 54 |
| IG2, N10000, p50 | 100 | 90 |
| **3-way interaction, detection success %** | | |
| **Experiment** | **PFI (top three features)** | **Three-way PFI** |
| IG3, N1000, p25 | 93 | 48 |
| IG3, N1000, p50 | 100 | 66 |
| IG3, N10000, p25 | 96 | 53 |
| IG3, N10000, p50 | 100 | 77 |
