# Supplementary material for: A comparison of methods for interpreting random forest models of genetic association in the presence of non-additive interactions
Source: BioData Min. 2021 Jan 29;14:9. doi: 10.1186/s13040-021-00243-0 (PMC7847145; doi:10.1186/s13040-021-00243-0)
Supplement: Supplementary file 4 — Additional file 4: Table S2. Spearman correlation coefficient for all features (A) and for the top 3 features (B). [file 13040_2021_243_MOESM4_ESM.docx]

Table S2. Spearman correlation coefficient for all features (A) and for the top 3 features (B)

*PFI -permutation feature importance, BIC – build-in coefficients, SHAP - shapley additive explanations,, IG- Information Gain, p25, p50 – percentage of cases, N – sample size after p-value cutoff, r - average spearman correlation coefficient*

| 1. **All features** | | | | | | | | |
| --- | --- | --- | --- | --- | --- | --- | --- | --- |
| **Sample size 1 000** | | | | | | | | |
| **IG** | **IG2** | | | | **IG3** | | | |
| **% of cases:** | **25 %** | | **50%** | | **25 %** | | **50%** | |
| **Metrics:** | **r** | **N, p<0.05** | **r** | **N, p<0.05** | **r** | **N, p<0.05** | **r** | **N, p<0.05** |
| PFI | 0.94 | 60 | 0.95 | 39 | 0.94 | 60 | 0.90 | 31 |
| BIC | 0.86 | 25 | 0.91 | 36 | 0.86 | 25 | 0.65 | 21 |
| SHAP | 0.92 | 44 | 0.93 | 55 | 0.92 | 44 | 0.95 | 47 |
| **Sample size 10000** | | | | | | | | |
| **IG** | **IG2** | | | | **IG3** | | | |
| **% of cases:** | **25 %** | | **50%** | | **25 %** | | **50%** | |
| **Metrics:** | **r** | **N, p<0.05** | **r** | **N, p<0.05** | **r** | **N, p<0.05** | **r** | **N, p<0.05** |
| PFI | 0.83 | 43 | 0.98 | 48 | 0.96 | 84 | 0.85 | 60 |
| BIC | 0.85 | 39 | 0.86 | 40 | 0.95 | 35 | 0.78 | 26 |
| SHAP | 0.91 | 68 | 0.91 | 59 | 0.94 | 52 | 0.88 | 65 |
| **B. Top 3 features** | | | | | | | | |
| **Sample size 1 000** | | | | | | | | |
| **IG** | **IG2** | | | | **IG3** | | | |
| **% of cases:** | **25 %** | | **50%** | | **25 %** | | **50%** | |
| **Metrics:** | **r** | **N, p<0.05** | **r** | **N, p<0.05** | **r** | **N, p<0.05** | **r** | **N, p<0.05** |
| PFI | 0.61 | 78 | 0.93 | 82 | 0.76 | 74 | 0.66 | 70 |
| BIC | 0.25 | 61 | 0.14 | 65 | -0.11 | 43 | -0.48 | 31 |
| SHAP | 0.63 | 75 | 0.86 | 85 | 0.57 | 47 | 0.59 | 64 |
| **Sample size 10000** | | | | | | | | |
| **IG** | **IG2** | | | | **IG3** | | | |
| **% of cases:** | **25 %** | | **50%** | | **25 %** | | **50%** | |
| **Metrics:** | **r** | **N, p<0.05** | **r** | **N, p<0.05** | **r** | **N, p<0.05** | **r** | **N, p<0.05** |
| PFI | 0.88 |  | 0.9 | 80 | 0.91 | 84 | 0.81 | 83 |
| BIC | 0.21 | 53 | 0.5 | 48 | 0.41 | 34 | -0.33 | 24 |
| SHAP | 0.72 | 85 | 0.87 | 75 | 0.53 | 59 | 0.67 | 66 |
